# COGNITIVE DECLINE PUBLIC HEALTH SURVEILLANCE: EXPERT-GUIDED COLLABORATION FOR BRFSS MODULE REVISION

**DOI:** 10.1093/geroni/igac059.2472

**Published:** 2022-12-20

**Authors:** John Shean, Benjamin Olivari

**Affiliations:** Alzheimer's Association, Chicago, Illinois, United States; CDC, Atlanta, Georgia, United States

## Abstract

The Behavioral Risk Factor Surveillance System (BRFSS) Cognitive Decline Module collects population level data on subjective cognitive decline (SCD) — self-reported difficulties in thinking or memory — which can be one of the earliest warning signs of dementia. Originally devised in 2009, the module undergoes periodic revision to ensure it remains current, the data collected are actionable, and to encourage uptake of the module by state health agencies. In 2021, the Alzheimer’s Association and the Centers for Disease Control and Prevention’s Alzheimer’s Disease Program formed an expert workgroup to examine the ongoing value and relevance of the module and propose any improvements. Workgroup members included researchers, chronic disease directors, epidemiologists, survey methodologists, policy analysts, and BRFSS coordinators. Using a consensus-building process, the workgroup utilized pre-meeting worksheets to identify areas of agreement and disagreement which informed key debates during subsequent meetings. Among many factors, the workgroup assessed the existing module for accuracy and utility. Discussion centered on aligning language and concepts contained within the module’s questions with current scientific research on SCD and how to accurately and adequately assess the associated burden SCD imposes. People living with cognitive impairment were consulted during the revision process to ensure phrasing reflected their lived experience. The result was a consensus set of suggested revisions to the Cognitive Decline Module, submitted to CDC for review and approval. The robust process, involving a variety of stakeholders and perspectives, can serve as an efficient and effective model for ensuring the longevity and usefulness of population health surveillance.

